# Distribution and pathological features of pancreatic, ampullary, biliary and duodenal cancers resected with pancreaticoduodenectomy

**DOI:** 10.1186/s12957-015-0498-5

**Published:** 2015-02-28

**Authors:** Manju D Chandrasegaram, Su C Chiam, John W Chen, Aisha Khalid, Murthy L Mittinty, Eu L Neo, Chuan P Tan, Paul M Dolan, Mark E Brooke-Smith, Harsh Kanhere, Chris S Worthley

**Affiliations:** HPB Surgery Unit, Royal Adelaide Hospital, North Terrace, Adelaide, SA 5000 Australia; Division of Surgery, School of Medicine, University of Adelaide, Adelaide, SA 5005 Australia; Flinders Medical Centre, Flinders Drive, Bedford Park, Adelaide, SA 5042 Australia; Flinders University, Sturt Rd, Bedford Park, Adelaide, SA 5042 Australia; School of Population Health, University of Adelaide, 178 North Terrace, Adelaide, SA 5005 Australia; HPB Surgery Unit, Queen Elizabeth Hospital, 28 Woodville Road, Adelaide, SA 5011 Australia

**Keywords:** Pancreatic cancer, Ampullary cancer, Biliary cancer, Duodenal cancer, Periampullary cancer, Margin, Pancreaticoduodenectomy, Histopathology, Whipple

## Abstract

**Background:**

Pancreatic cancer (PC) has the worst survival of all periampullary cancers. This may relate to histopathological differences between pancreatic cancers and other periampullary cancers. Our aim was to examine the distribution and histopathologic features of pancreatic, ampullary, biliary and duodenal cancers resected with a pancreaticoduodenectomy (PD) and to examine local trends of periampullary cancers resected with a PD.

**Methods:**

A retrospective review of PD between January 2000 and December 2012 at a public metropolitan database was performed. The institutional ethics committee approved this study.

**Results:**

There were 142 PDs during the study period, of which 70 cases were pre-2010 and 72 post-2010, corresponding to a recent increase in the number of cases. Of the 142 cases, 116 were for periampullary cancers. There were also proportionately more PD for PC (26/60, 43% pre-2010 vs 39/56, 70% post-2010, *P* = 0.005). There were 65/116 (56%) pancreatic, 29/116 (25%), ampullary, 17/116 (15%) biliary and 5/116 (4%) duodenal cancers. Nodal involvement occurred more frequently in PC (78%) compared to ampullary (59%), biliary (47%) and duodenal cancers (20%), *P* = 0.002. Perineural invasion was also more frequent in PC (74%) compared to ampullary (34%), biliary (59%) and duodenal cancers (20%), *P* = 0.002. Microvascular invasion was seen in 57% pancreatic, 38% ampullary, 41% biliary and 20% duodenal cancers, *P* = 0.222. Overall, clear margins (R0) were achieved in fewer PC 41/65 (63%) compared to ampullary 27/29 (93%; *P* = 0.003) and biliary cancers 16/17 (94%; *P* = 0.014).

**Conclusions:**

This study highlights that almost half of PD was performed for cancers other than PC, mainly ampullary and biliary cancers. The volume of PD has increased in recent years with an increased proportion being for PC. PC had higher rates of nodal and perineural invasion compared to ampullary, biliary and duodenal cancers.

## Background

Pancreaticoduodenectomies (PDs) are performed for resectable periampullary cancers, which are pancreatic, ampullary, biliary and duodenal cancers. The proportion of these cancers resected with a PD in contemporary series is variable. In a recent large three-decade study of 2,564 resected periampullary cancers, by He *et al*., the distribution of resected pancreatic, ampullary, biliary and duodenal carcinomas were 66%, 16%, 12%, and 6%, respectively [1]. An Australian series by Chen *et al*. of 96 patients undergoing PD for malignancy found the proportion of resected pancreatic, ampullary, biliary and duodenal cancers were 63%, 15%, 9%, and 9%, respectively [2].

Ampullary cancers were historically thought to be an uncommon tumour amongst patients undergoing PD. Given that approximately 20% of pancreatic cancers are amenable to resection compared to over 80% of ampullary cancers, ampullary cancers represent a significant proportion of patients undergoing a pancreaticoduodenectomy [3]. A recent large Chinese series by Chen *et al*. of 501 periampullary cancers found that ampullary carcinomas represented the majority of cancers subjected to a PD at 50%, followed by pancreatic cancer 34%, biliary cancer 10% and duodenal cancer 5% [4]. This high incidence of ampullary cancers in the Chinese series may relate to geographic differences in the epidemiology of these cancers.

Pancreatic cancer has a poorer survival compared to biliary and ampullary cancers and although they are different histologically, it is unclear whether underlying tumour behaviour or invasiveness alone contributes to these differences in survival [5]. Despite the poor survival with pancreatic cancer, nearly 20% of patients with resected pancreatic cancer have a chance of long-term survival [6]. Several studies have shown a higher incidence of nodal, neural and vascular invasion in pancreatic cancers compared to ampullary cancers, which may relate to the differences seen in survival [7].

The aim of this study was to examine the distribution and histopathologic features of pancreatic, ampullary, biliary and duodenal cancers resected by PD and referred for analysis to a single large public metropolitan laboratory, and also to determine whether there were changes in trends over time.

## Methods

A retrospective review of PD histopathology reports was performed between January 2000 and December 2012 at the South Australian (SA) Pathology database at the Royal Adelaide Hospital. The institutional ethics committee approved this study. A search of the SA Pathology database was performed to identify all consecutive PD reports during the study period.

The database included demographic data and histopathological reports.

### Demographics

The age, gender distribution of the study population and their cancer subgroups were analysed.

### Pathological characteristics

Cancers were identified and subgrouped as pancreatic, ampullary, biliary and duodenal cancer to identify the proportions of each cancer resected with a PD. Tumour size, nodal status, total number of nodes resected, presence of microvascular or neural invasion was assessed for each of the tumour subgroups.

### Margin assessment

Microscopic margin (R1) involvement was defined as the presence of microscopic tumour at the mobilization or transection margins of the surgical specimen as defined by the International Union Against Cancer [8]. The anterior and posterior margins were the margins located anteriorly and posteriorly as referenced by the pancreas when lying in its usual anatomic location and are synonymous with the ‘mobilization margins’. The medial margin or the superior mesenteric vessel margin is that margin bordered by the uncinate process alongside the superior mesenteric vessels. The pancreatic neck margin is the transection margin of the pancreas at the neck of the pancreas anterior to the porto-mesenteric vein confluence or to the left of this. The medial margin and the pancreatic neck margin are also known as the ‘transection margins’.

The proportion of patients with clear margins before and after 2010 was performed as a subset analysis to assess if these were any different with more recent surgical practice.

### Statistical methods

Statistical methods used in this study are mostly limited to simple descriptive methods such as chi-squared test for testing both the 2 × 2 associations and trend test for proportions [9,10]. Due to the small numbers in three- and four-way classification, multivariate analysis was not performed. However, simple two-way cross-tabulation between cancer type and nodal disease, perineural invasion and vascular invasion were performed to assess for association.

Multinomial logistic regression between cancer type and age was carried out to evaluate the age-adjusted effect on the risk of cancer type. Multinomial regression was used since the outcome was categorical.

All statistical analyses were performed in STATA version 13 [11]. All *P* values calculated were two-tailed; the alpha level of significance was set at 0.05.

## Results

### Study population

There were 142 PD patients during the study period, of which 126 were referred from public hospitals (70 from Royal Adelaide Hospital, 40 from Queen Elizabeth Hospital and 16 from Lyell McEwin Health Service), whilst 16 were from private institutions. Seventy PDs were performed between 2000 and 2009 and 72 between 2010 and 2012. There were 116 PDs performed for pancreatic, ampullary, biliary and duodenal adenocarcinoma and 6 for neuroendocrine and 5 for intraductal mucinous tumours. Of these 116 resections for adenocarcinoma, almost half, 56 (48%), was performed between the periods 2010 to 2012.

### Demographics

Of the 116 PDs identified above, there were 67 males (58%) and 49 females (42%) aged between 26 and 86 years. The most common pathology for which a PD was performed was pancreatic cancer (65/116, 56%), followed by ampullary cancer (29/116, 25%), biliary cancer (17/116, 15%) and duodenal cancer (5/116, 4%) (Table 1).

**Table 1:** **Distribution of tumour pathology, size, nodal, vascular and neural invasion in pancreaticoduodenectomy specimens**

**Tumour type**	**n (%)**	**Tumour size median (mm)**	**Nodal involvement n (%)**	**Microvascular invasion**	**Neural invasion**
Pancreatic	65 (56%)	35	51 (78%)	37 (57%) 5 S; 9 U	48 (74%) 4 S; 5 U
Ampullary	29 (25%)	23	17 (59%)	11 (38%) 9 U	10 (34%) 7 U
Biliary	17 (15%)	27	8 (47%)	7 (41%) 2 S; 1 U	10 (59%) 1 S; 1 U
Duodenal	5 (4%)	30	1 (20%)	1 (20%)	1 (20%)

S, suspicious; U, unreported.

The median age of all the patients undergoing a PD for a cancer was 67 years (range 26 to 86). Patients with pancreatic cancer had a median age of 64 years (range 26 to 81), those with ampullary cancer had a median age of 69 years (range 42 to 86), biliary cancer median age 66 years (range 55 to 85) and duodenal cancer median age of 77 years (range 51 to 84).

### Pancreatic cancer

There were 65/116 (56%) pancreatic cancers. The median size of these cancers was 35 mm. The vast majority of these tumours were node positive (51/65, 78%). The median number of nodes harvested during a PD was 12 (range 1 to 27).

A clear resection margin was achieved in 41/65 (63%), with microscopic disease at the margin seen in 24/65 (37%). The anterior margin was involved in 6/24 patients, posterior margin in 5/24 patients, medial margin in 12/24 patients, and the neck margin in 5/24 patients (Table 2).

**Table 2:** **Resection margin status by tumour type**

**Margin status**	**Pancreatic (n = 65)**	**Ampullary (n = 29)**	**Biliary (n = 17)**	**Duodenal (n = 5)**
Clear margin	41 (63%)	27 (93%)	16 (94%)	4 (80 %)
Positive margin	24 (37%)	2 (7%)	1 (6%)	1 (20%)
Anterior margin	6	0	1	1
Posterior margin	5	1	0	1
Medial/SMA margin	12	1	1	0
Pancreatic neck margin	5	0	0	0
R2 (macroscopic margin involvement)	3	0	0	0

Prior to 2010, a clear resection margin was achieved in 12/26 (46%) patients, and after 2010, a clear resection margin was achieved in 26/39 (67%) patients. There was evidence of microvascular invasion in 37/65 (57%) patients and perineural invasion in 48/65 (74%) patients.

There was no evidence of nodal, microvascular or perineural involvement in only 2/65 patients with pancreatic cancer.

### Ampullary cancers

There were 29/114 (25%) ampullary cancers. The median size of these cancers was 23 mm. Approximately half of these tumours were node positive (17/29, 59%). The median number of nodes harvested was 7.5 (range 1 to 26).

A clear resection margin was achieved in 27/29 (93%), with microscopic disease at the margin seen in 2/29 (7%). The two patients with involved margin had involvement on the posterior margin in one and the medial margin in the other.

Prior to 2010, a clear resection margin was achieved in 18/20 (90%), and after 2010, a clear resection margin was achieved in all nine patients (100%).

There was evidence of microvascular invasion in 11/29 (38%) patients and perineural invasion in 10/29 (34%) patients.

There was no evidence of nodal, microvascular or perineural involvement in only 5/29 patients with ampullary cancer.

### Biliary cancers

There were 17/114 (15%) biliary cancers. The median size of these cancers was 27 mm. Approximately half of these tumours were node positive (8/17, 47%) The median number of nodes harvested was 12 (range 4 to 30).

A clear resection margin was achieved in 16/17 (94%), with microscopic disease at the margin seen in one patient who had involvement of both the anterior and medial margins.

Prior to 2010, a clear resection margin was achieved in 9/11 (82%), and after 2010, a clear resection margin was achieved in all six patients (100%).

There was evidence of microvascular invasion in 7/17 (41%) patients and perineural invasion in 10/17 (59%) patients.

There was no evidence of nodal, microvascular or perineural involvement in only 1/17 patient with biliary cancer.

### Duodenal cancers

There were 5/114 (4%) duodenal cancers. The median size of these cancers was 30 mm. One of these tumours was node positive. The median number of nodes harvested was 9 (range 2 to 22).

A clear resection margin was achieved in 4/5 (80%) with microscopic disease at the margin seen in 1/4 (25%) patient. This patient had involvement of both the anterior and posterior margins.

Prior to 2010, a clear resection margin was achieved in 2/3 (67%), and after 2010, a clear resection margin was achieved in 2/2 (100%) patients.

There was evidence of microvascular invasion in 1/5 (20%) patient, and perineural invasion in 1/5 patient (20%).

There was no evidence of nodal, microvascular or perineural involvement in 4/5 patients with duodenal cancer.

### Cancer type and histopathological features

There was statistically significant association between cancer type and the incidence of nodal disease (chi-square, *P* value = 0.002) and the incidence of perineural invasion (chi-square, *P* value = 0.002). There was no significant association between cancer type and the incidence of microvascular invasion (chi-square, *P* value = 0.222).

### Trends before and after 2010

In the 3 years following 2010, there was an increase in the annual number of PD compared to the preceding 10 years - 70 (7 PD/year) vs 72 (24 PD/year). A chi-square test for trend in proportions was performed to test if the change was statistically significant. Results infer that the trend was statistically significant (*P* value for trend being 0.002), thus indicating an increase in PD.

The proportion of patients with pancreatic cancer following PD also increased significantly from 26/60 (43%) pre-2010 to 39/56 (70%), *P* = 0.005. Interesting, there was an improvement in R0 resection rates in the 3 years following 2010. A clear margin was achieved in 46% of patients prior to 2010 and 67% of patients after 2010.

Multinomial logistic regression between cancer type and age showed that the association between age and type of cancer was not statistically significant (*P* values between 0.22 and 0.36). However, this must be interpreted with caution due to the low number of cases within the cancer types.

## Discussion

In our cohort of 116 patients, PD was performed for pancreatic cancer in 56% of patients and ampullary cancer in 25%. Whilst the proportion of resected ampullary cancers is higher than contemporary series from the US, it is lower than the Chinese series [4]. Pancreatic cancers in this series had a higher incidence of nodal involvement, microvascular invasion and perineural invasion.

Due to the study being conducted through a pathology database at a specific hospital site, not all patients would have been captured from all of the other sites. However, it was apparent that there was an overall increase in the proportion of patients with PD for pancreatic cancer in the later part of our series.

The advent of synoptic reporting will allow us to consistently assess pathological features such as nodal, microvascular and perineural invasion in different cancers and analyse their prognostic relevance. Earlier reports in this series had inconsistent reporting of perineural and microvascular invasion, which improved with the introduction of synoptic reporting.

Ampullary cancers represented a significant proportion of cancers (29/116, 25%) that were subjected to a PD in our series, higher than the 16% reported by He *et al*. and 23% reported by Hatzaras *et al*.

Fifty-nine percent of patients with ampullary carcinoma in our series had lymph node involvement compared to 47% in the series by Brown *et al*. They showed that the 5-year survival with ampullary carcinoma was 78% without nodal involvement and 25% with nodal involvement [12]. Survival with ampullary cancer is also diminished in the presence of vascular invasion and neural invasion [13].

Patients with ampullary cancers in our series were slightly older than those with pancreatic cancer. In a historical cohort (1981 to 1997) of ampullary tumours from our institution, 13/20 patients with ampullary cancer in that period underwent a PD, and this was the recommended treatment in fit patients [14]. Yeh *et al*. analysed their survival of patients under and over the age of 75 undergoing a PD for ampullary cancers and found that there was no difference in survival or outcomes between the two age groups [15]. This supports resection for ampullary cancers in fit elderly patients.

Pancreatic cancers have the worst survival compared to other periampullary cancers [16]. In the recent Johns Hopkins series by He *et al*., pancreatic cancer had the worst survival (median survival 19 months, 5-year overall survival (OS) 18%), followed by bile duct cancer (median survival 23 months, 5-year OS 27%), ampullary cancer (median survival 47 months, 5-year OS 45%) and duodenal cancer (median survival 54 months, 5-year OS 49%) [1].

In our series, patients with pancreatic cancer had the highest incidence of nodal involvement (78%), perineural invasion (74%) and microvascular invasion (57%) all of which have been shown to be poor prognostic features. The higher incidence of nodal involvement in pancreatic compared to ampullary cancers has been demonstrated in several series [1,7,13,17]. In the large series of 1,175 resected pancreatic cancers by Winter *et al*., the incidence of nodal involvement (78%) and vascular involvement (53%) was similar to our series; however, the incidence of perineural invasion was much higher in their series at 91% [18]. In a more recently published series of 1,822 resected pancreatic cancers by Mayo *et al*. there was a 69% incidence of perineural invasion [19]. In addition to providing an alternate route for metastatic spread, perineural invasion is involved in pain generation in patients with pancreatic cancer [20].

Pancreatic cancers had a 37% incidence of microscopic margin involvement, which was disproportionately higher than the 6% to 7% seen in both ampullary and biliary cancers. Our positive resection margin rates are comparable to an Australian statewide series by Speer *et al*. [21]. In our series, a clear margin was achieved in 46% of patients prior to 2010 and 67% of patients after 2010. Changes and improvement in surgical technique may have accounted for this difference; however, this study was not designed to explore this.

In the Australian series by Chen *et al*. the combined presence of both perineural and lymphovascular invasion following PD reduced the 5-year-survival from 71 % (when both were absent) to 16% (when both were present) [2]. In our series, only 2/65 (3%) patients with pancreatic cancer and 1/17 with biliary cancer had no evidence of nodal, microvascular or perineural invasion. In contrast, 5/29 (17%) patients with ampullary cancer and 4/5 (80%) patients with duodenal cancer had no nodal, microvascular or perineural invasion. A follow-up study on survival will evaluate this further.

It may well be that the poorer survival seen in patients with pancreatic cancer as opposed to other subgroups undergoing a PD lies in the high incidence of lymphovascular and perineural invasion compared to ampullary cancers as seen in this study. This would be fitting with Whipple’s hypothesis that pancreatic cancers have a worse prognosis compared to ampullary cancers because they exhibit early lymphovascular and perineural invasion [3,22].

The definition of a positive margin in our series was the presence of cancer to the mobilization surface of the pancreas (anterior and posterior) or the transection surface (pancreatic neck or medial margin). There are variable definitions as to what constitutes a positive margin, and a large range of figures for margin positivity exists depending on how stringent the pathological definitions and assessments are [23,24].

In the large series by Winter *et al*. of 1,175 resected pancreatic cancers, 42% had a positive margin. Margin status has been shown to relate to survival outcomes. Yeo *et al*. showed in their series of 201 patients that the actuarial 5-year survival in those with a negative margin was 26% (median survival 18 months) compared to those with a positive margin 8% (median survival 10 months) [25]. Whilst margin positivity appears to be critically important, not all margins may have a similar impact or bearing on survival. Delpero *et al*. reported that whilst a positive SMA or SMV margin had a significant impact on progression-free survival, a positive posterior margin had no impact [26].

A higher incidence of margin positivity is reported depending on the definition used such as the British Royal College of Pathologists (RCPath) and the Royal College of Pathologists Australia (RCPA) that regards tumour ≤1 mm from a pancreatic resection margin as an involved margin [27,28]. A positive margin using this definition was seen in 109/148 (74%) of patients in the study by Jamieson *et al*. [29]. In the study by Hartwig *et al*., the positive margin rate was 64% using this revised definition [30].

In our study, of the 24 patients with a positive margin, half was positive at the medial margin. There were five positive pancreatic neck margins, and frozen section of the pancreatic neck margin may reduce this incidence. In a study by Pang *et al*. of 101 frozen sections of the pancreatic neck margin, 19 (19%) were positive. Of these 19, re-resection enabled 16 to have a negative neck margin, but only seven had no other margin involvement. Although the authors found that frozen section improved R0 rate by 7%, this did not improve survival [31].

In an era where our understanding of the molecular basis of disease is increasing, each cancer needs to be recognised as a distinct entity with its own histomolecular characteristics. This will enable us to treat each cancer distinctly as a unique and separate entity. An obvious example is that whilst pancreatic cancers invariably have KRAS mutation in over 90% of these cancers and a higher incidence of DPC4 inactivation of up to 55%, this is not seen to the same degree in ampullary or biliary cancers [32-37]. This has significant bearing on future clinical trials by targeting discrete molecular and genetic aberrancies within these cancers [38]. KRAS mutation is seen in up to 17% of bile duct cancers and up to 52% of ampullary cancers [39,40].

## Conclusions

This study highlights that almost half of PD in a single government-administered large metropolitan database are performed for cancers other than pancreatic cancer, mainly ampullary and biliary cancers. These cancers need to gain separate attention to pancreatic cancers in clinical trials as they represent a large proportion of cancers resected with a PD. The volume of PD has increased in recent years with an increased proportion being for PC. PC had higher rates of nodal and perineural invasion compared to other periampullary cancers.
